# Aerosol Chemistry over a High Altitude Station at Northeastern Himalayas, India

**DOI:** 10.1371/journal.pone.0011122

**Published:** 2010-06-16

**Authors:** Abhijit Chatterjee, Anandamay Adak, Ajay K. Singh, Manoj K. Srivastava, Sanjay K. Ghosh, Suresh Tiwari, Panuganti C. S. Devara, Sibaji Raha

**Affiliations:** 1 Environmental Sciences Section, Bose Institute, Kolkata, India; 2 Center for Astroparticle Physics and Space Science, Bose Institute, Kolkata and Darjeeling, India; 3 Department of Geophysics, Banaras Hindu University, Varanasi, India; 4 Department of Physics, Bose Institute, Kolkata, India; 5 Indian Institute of Tropical Meteorology, New Delhi, India; 6 Indian Institute of Tropical Meteorology, Pune, India; Universidade de Vigo, Spain

## Abstract

**Background:**

There is an urgent need for an improved understanding of the sources, distributions and properties of atmospheric aerosol in order to control the atmospheric pollution over northeastern Himalayas where rising anthropogenic interferences from rapid urbanization and development is becoming an increasing concern.

**Methodology/Principal Findings:**

An extensive aerosol sampling program was conducted in Darjeeling (altitude ∼2200 meter above sea level (masl), latitude 27°01′N and longitude 88°15′E), a high altitude station in northeastern Himalayas, during January–December 2005. Samples were collected using a respirable dust sampler and a fine dust sampler simultaneously. Ion chromatograph was used to analyze the water soluble ionic species of aerosol. The average concentrations of fine and coarse mode aerosol were found to be 29.5±20.8 µg m^−3^ and 19.6±11.1 µg m^−3^ respectively. Fine mode aerosol dominated during dry seasons and coarse mode aerosol dominated during monsoon. Nitrate existed as NH_4_NO_3_ in fine mode aerosol during winter and as NaNO_3_ in coarse mode aerosol during monsoon. Gas phase photochemical oxidation of SO_2_ during premonsoon and aqueous phase oxidation during winter and postmonsoon were the major pathways for the formation of SO_4_^2−^ in the atmosphere. Long range transport of dust aerosol from arid regions of western India was observed during premonsoon. The acidity of fine mode aerosol was higher in dry seasons compared to monsoon whereas the coarse mode acidity was higher in monsoon compared to dry seasons. Biomass burning, vehicular emissions and dust particles were the major types of aerosol from local and continental regions whereas sea salt particles were the major types of aerosol from marine source regions.

**Conclusions/Significance:**

The year-long data presented in this paper provide substantial improvements to the heretofore poor knowledge regarding aerosol chemistry over northeastern Himalayas, and should be useful to policy makers in making control strategies.

## Introduction

Atmospheric aerosol is linked to visibility reduction, adverse health effects and heat balance of the Earth, directly by reflecting and absorbing solar radiation and indirectly by influencing the properties and cloud processes and, possibly, by changing the heterogeneous chemistry of reactive greenhouse gases [1]. The combined global radiative forcing due to increases in major greenhouse gases (CO_2_, CH_4_ and N_2_O) is +2.3 Wm^−2^. Anthropogenic contributions to aerosols (primarily sulphate, organic carbon, nitrate and dust) together produce a cooling effect, with a total direct radiative forcing of −0.5 Wm^−2^ and an indirect cloud albedo forcing of −0.7 Wm^−2^ [1]. Thus aerosols compensate by ∼50% for the mean global radiative forcing due to greenhouse gases warming. The large range of uncertainty in estimating the aerosol forcing reflects the poor state of knowledge regarding the sources, distributions and properties of atmospheric aerosol.

Increasing pollutant emissions associated with the fast-growing economies of southeastern Asian countries have led to the progressive increase of aerosol concentrations above the natural background [2]. Satellite observations have shown that the light-absorbing aerosol hazes (which is about 3–5 mm thick) over India intensify over the Thar desert and the polluted Indo-Gangetic plain (IGP). The IGP has a sharp boundary to the north, where the Himalayas act as a barrier, extending thousands of miles southward and over the north Indian ocean [3], [4]. Aerosol rich boundary layer air can be transported to higher altitudes by valley breezes on the Himalayan slopes [2].

The transport of optically-active aerosol to the higher Himalayas is a matter of concern, since most of the glaciers in the region have been retreating since 1850 [5] with increasing melting rates, and are in danger of completely disappearing in the next decades [6]. If the retreat of the Himalayan glaciers continues unabated, it will exacerbate the water stress in northern India, especially during the dry season [7]. The rising anthropogenic interferences from rapid urbanization and development in the Himalayas affect both the landscape and the atmospheric environments and are the causes of increasing concern [8], [9].

A short-term sampling program in the Nguzompa glacier basin near Mt. Everest [10], a two-week sampling project in Hidden Valley in the Himalayas of western Nepal [11], a year-long sampling of atmospheric aerosol at a remote Himalayan site and a rural Middle-Mountain site in Nepal [12], a study on the effect of mineral dust and carbonaceous species on the aerosol composition in Nepal Himalayas [13] and a study on the seasonal variation of total suspended particulates (TSP) in Manali, northwestern Himalayan range [14] are the major research studies carried out in the Nepal Himalayan and in other northwestern Himalayan sites. But as far as the northeastern Himalayas are concerned, the region still lacks systematic studies focused on chemical characterization of aerosols.

A strong seasonal variation in aerosol chemistry is expected in this northeastern Himalayan region. During premonsoon and summer months, due to enhanced convection, aerosols are lofted to elevated altitudes in the troposphere. Together with the westerly premonsoon winds, enhanced convection and the steep pressure gradient across the Himalayan-Gangetic region steer aerosols aloft. With the onset of rainy season (the Arabian Sea and Bay of Bengal branches of the South West summer monsoon), the heavy dust loading significantly diminishes due to aerosol washout from the atmosphere and enhances the loading of sea salt aerosols to a significant level. During winter and postmonsoon, northeasterly winds from the subcontinent bring anthropogenic aerosols over the Himalayan region. In addition to that, massive biomass burning during winter also plays a role in the loading of anthropogenic aerosols over northeastern Himalayas. These distinctly different seasonal behaviors of aerosol in northeastern Himalayas prompted us to make a year-long study on the formation and distribution of atmospheric aerosols over Darjeeling, a high altitude station in northeastern Himalayas.

In order to understand the seasonal nature of the predominant water soluble ionic species in fine (aerodynamic diameter less than 2.5 µm) and coarse mode (aerodynamic diameter more than 2.5 µm) aerosol a year-long aerosol sampling was done during January–December 2005. This study presents the possible formation mechanisms of secondary ionic species in different seasons, distribution of primary ionic species, long range transport of dust aerosol and the interaction between transported marine aerosol and locally generated anthropogenic aerosol. Finally, an attempt was made to find out the possible types of aerosols from different source regions.

The primary focus of this study was to determine the relative contribution of natural and anthropogenic components on the total aerosol loading and their distribution between fine and coarse mode aerosols at a high altitude hill station in northeastern Himalayas which could provide the scientific basis for controlling atmospheric pollution over this geo-politically and environmentally important region.

### 1. Site description

Darjeeling is one of the most popular tourist hill-stations in eastern India with a population of ∼100,000. The overall areas of the Darjeeling district and Darjeeling Township are about 1200 and 11.44 squared kilometers, respectively. Darjeeling Township is located at an average altitude of ∼2000 meter above sea level (masl) and surrounded by different types of topography of the lower-eastern-Himalayas. The southern region comprises the marshy low-lying area at an average height of ∼100–300 masl. The apex is formed by the Phalut ridge (altitude of 3800 masl) at the border between Nepal and India. The eastern frontier lies along two rivers, locally called Tista and Rangeet.

**Figure 1:**
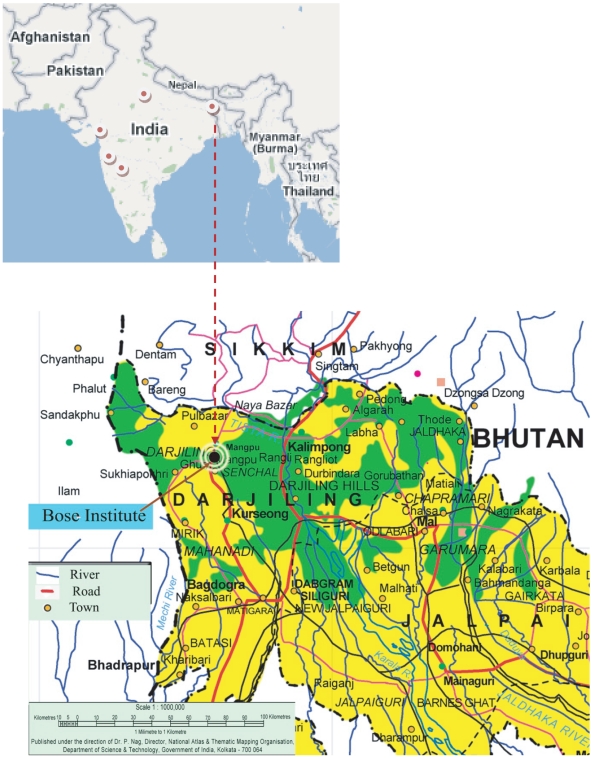
Map of the sampling station.

The sampling site (shown in Figure 1) is located on the terrace of a three-storied building on our institute premises. This site (latitude: 27°01′N, longitude: 88°15′E with an altitude of 2194 masl) is at an altitude of about 200 m above the main township and is a remote area compared to the main township with a limited number of residential houses and forested areas dominated by juniper and varieties of pine in the immediate vicinity of the observatory. The closest street with significant road traffic is about 200 m away from the study site. The area, within a radius of ∼10 km, is occupied by several major and minor tea processing units operated by furnace oil and coal and several tea gardens where several ammoniated fertilizers are used. Wood and biomass burning in the nearby villages, automobile exhaust (mainly tourist vehicles) throughout the year and the exhaust from the “Toy Train” (Darjeeling Himalayan Railway), which is enlisted as an UN (United Nations) world-heritage and still runs on coal as its fuel, are the major sources of air pollution at this hill station.

### 2. Prevailing meteorology

**Figure 2:**
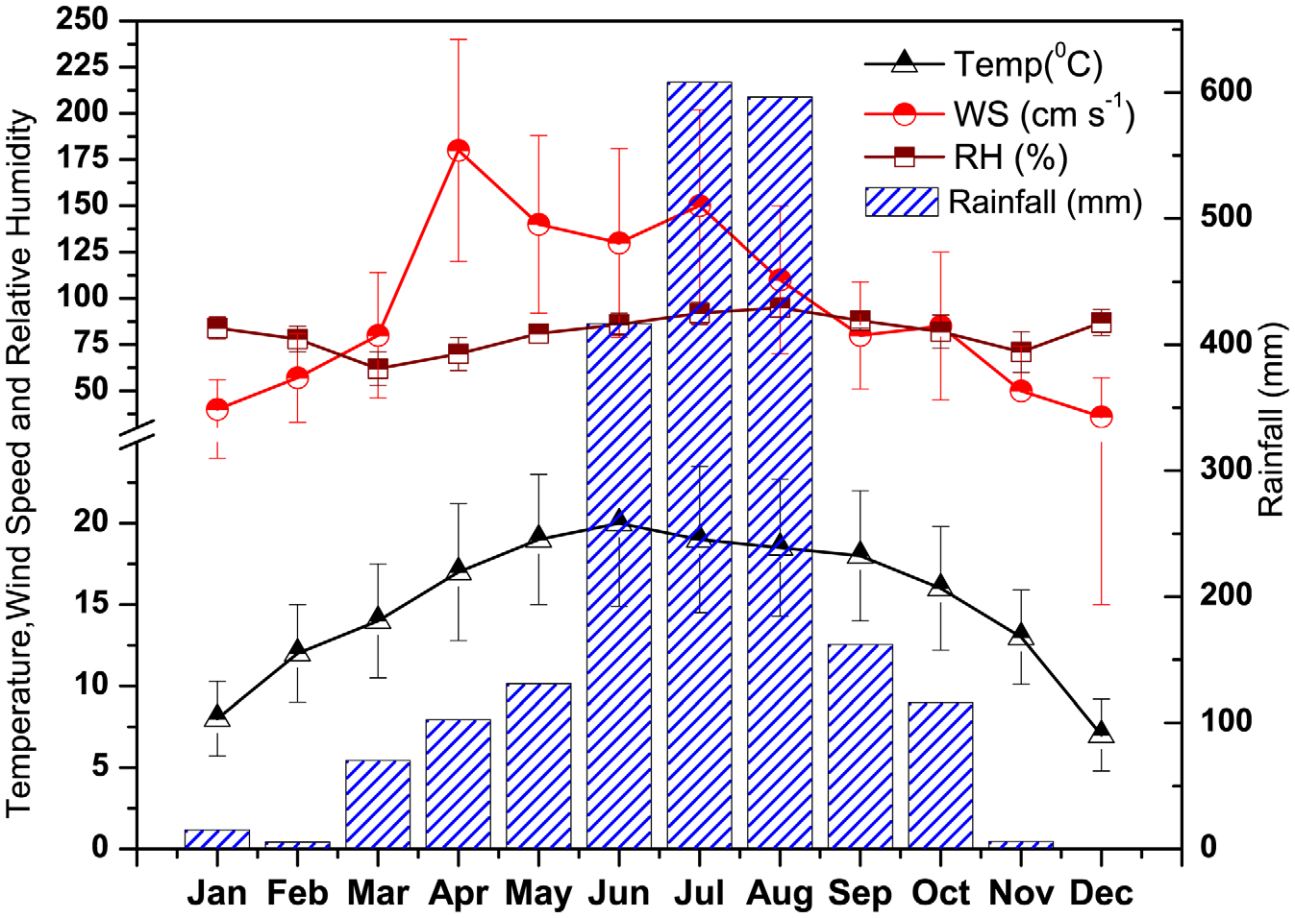
Seasonal variations of temperature (Temp), wind speed (WS), relative humidity (RH) and rainfall over Darjeeling.

**Figure 3:**
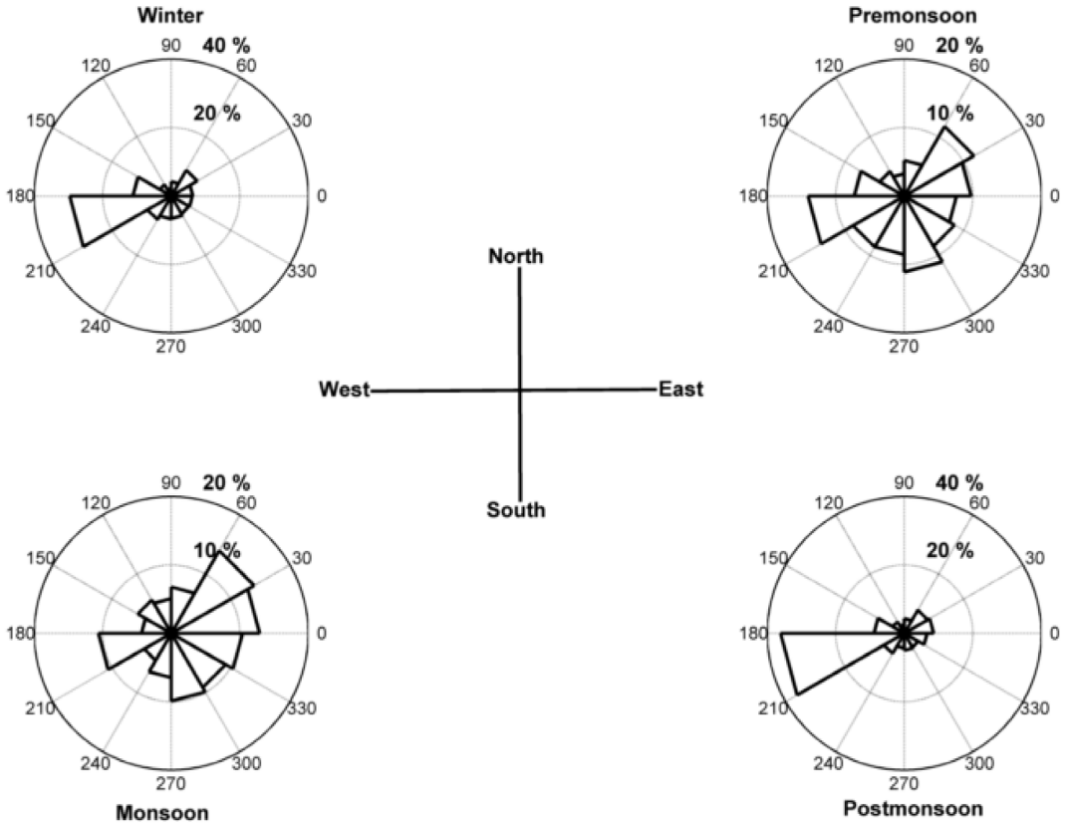
Rose diagram of surface wind speed and direction over the entire study period in Darjeeling.

**Figure 4:**
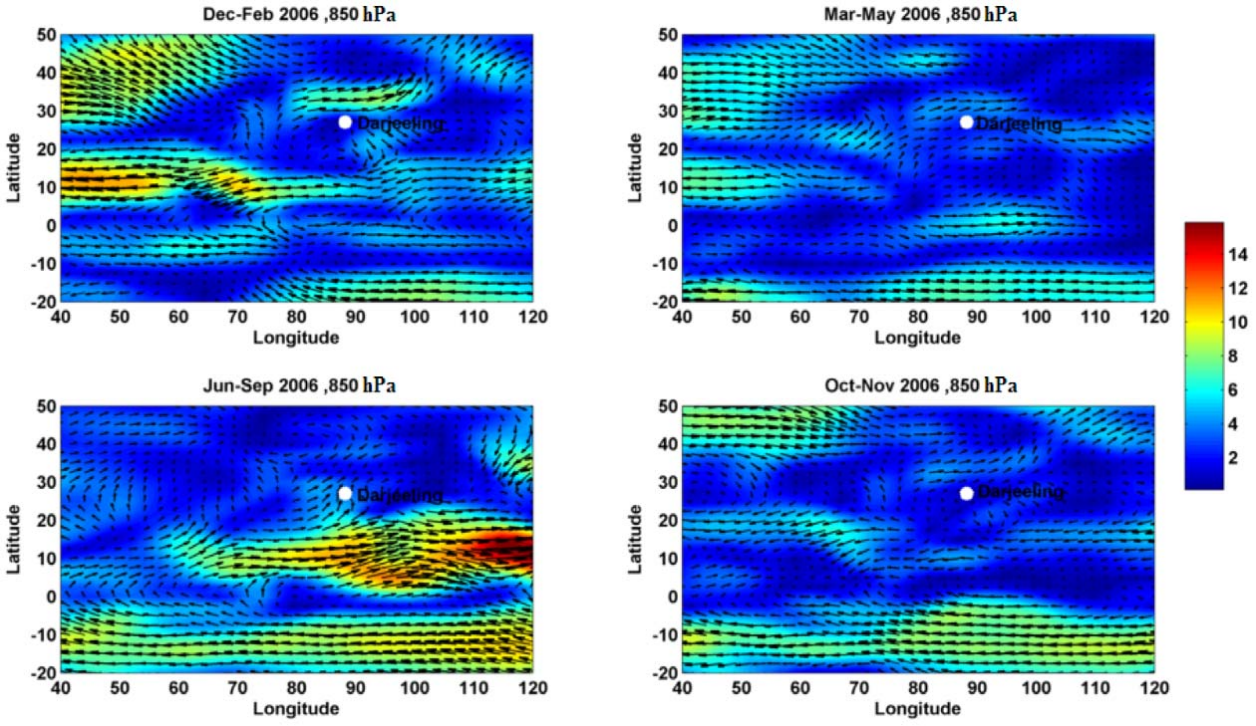
Mean wind at 850 hPa level obtained from NCEP/NCAR reanalysis during different seasons over Darjeeling.

The monthly variations of surface meteorological parameters like temperature (°C), relative humidity (%), wind speed (m s^−1^) along with total rainfall (mm) over the entire study period (Jan–Dec) are shown in Figure 2 and the surface wind directions presented seasonally in different seasons, namely, winter (Dec–Feb), premonsoon (Mar–May), monsoon (Jun–Sep) and postmonsoon (Oct–Nov) in Figure 3. The average temperature was found to be 15±4°C with minimum of 7°C during December and maximum of 20°C during June. In general, the relative humidity was high across the whole study period with an average of 81%. The dry season (Jan–May, Oct–Dec) remained moderately dry with an average relative humidity of 76% compared to the wet season with an average relative humidity of 91%. The total rainfall during the entire study period (Jan–Dec) was found to be 2220 mm, 80% of which was during southwest monsoon (1783 mm) with scanty or no rainfall during winter (20 mm) and premonsoon (304 mm). The surface wind pattern during winter was mainly easterly and northeasterly with average speed of 0.84 m s^−1^ and during monsoon it was mainly southwesterly with an average speed of 1.18 m s^−1^. In order to know the wind pattern variations in different seasons, the monthly mean wind vectors (at 850 hPa level) for four different seasons; winter (Dec–Feb), premonsoon (Mar–May), monsoon (Jun–Sep) and postmonsoon (Oct–Nov) are shown in Figure 4, for the region covering equator to 40°N and 40–130°E. The NCEP/NCAR reanalysis data clearly show the contrasting wind patterns between winter and monsoon whereas premonsoon and postmonsoon represents the transition phase in the circulation patterns. Winter shows the weak northeasterly wind from the continental area covering densely populated cities including semi arid regions whereas monsoon shows strong southwesterly wind originating from Arabian Sea. These distinctly different wind fields impart extreme temporal variability in aerosol characteristics.

## Methods

### 1. Collection of aerosol samples and determination of mass concentration

For the collection of total respirable suspended particulate matter (aerodynamic diameter less than 10 µm) from ambient air, a respirable dust sampler was used. The sampler (model APM 460BL) was manufactured by Envirotech Instrument Pvt Ltd, which pioneered the development of indigenous air monitoring instruments all over India. The sampler collected the samples with a flow rate of 1.4 m^3^ min^−1^. The sampler was fitted with a cyclone, which was used for fractioning the dust into two fractions. Total respirable particulate matter was collected on the filter paper (EPM 2000 filter paper from Whatman of 8″×10″ dimension) while particulate matter of aerodynamic diameter more than 10 µm was collected in a cup placed under the cyclone.

For collection of fine particulate matter or fine mode aerosol, a fine dust sampler was used from the same company (model APM 550). The flow rate was 1 m^3^ hr^−1^. After entering the air particles through the inlet of the sampler the coarse particulate matter (aerodynamic diameter more than 2.5 µm) was removed using a GF/A (Glass Fiber) filter paper of 37 mm diameter immersed in silicone oil, used as an impaction surface. The impaction surface was placed above the main aerosol collection filter. The fine particles were collected on a PTFE filter paper of 47 mm diameter.

On average, an aerosol sample (both for fine and total respirable particulate matter) was collected on every 3^rd^ day during dry seasons (Jan–May, Oct–Dec) and every 4^th^ day during monsoon (Jun–Sep). Thus a total of 111 samples were collected (81 during dry season and 30 during monsoon). Although collection of samples on daily basis would be better in carrying out this kind of aerosol study, collection of samples of more than 111 were beyond our scope. Each sampling was started at 0900 hrs (local time) and run for ∼24 hrs. Both the samplers were placed on the terrace of a three-storied building (∼15 m above ground level) on our institute premises.

The mass concentration of aerosol was determined by the gravimetric measurement. The filters were placed in desiccators for ∼24 hrs before and after the sampling to remove the absorbed water and weighed in a controlled environment chamber after taking the filters out of the desiccators before and after the sampling using a semi-micro balance (Sartorius, Model ME 235 P). The aerosol mass (µg) was determined by the differences between initial and final weight of the filter and the concentration (µg m^−3^) was determined dividing the aerosol mass by total volume of air (m^3^).

### 2. Meteorological parameters

The meteorological parameters were recorded with the help of an automatic weather station of Lawrence & Mayo (Model: AWS-PC) and all the data were run by LYNX-software of version V0007. The weather station was run continuously and the data were recorded at the interval of half an hour throughout the year covering all the sampling events. The weather station was equipped with a tower and all the sensors of wind speed and its direction, relative humidity (RH) and temperature were fitted with that tower at a height of 15 m from the ground level. The rainfall data was obtained from Indian Meteorological Department, India.

### 3. Chemical analysis

For the analysis of water soluble ions, chromatographic separation method was used [15]. One-half of the filters were soaked in 20 ml Milli-Q water (18.2 MΩ resistivity) for ∼30 min. and ultrasonicated for 20 min. The solutions were made up to known volume (100 ml) using Milli-Q water. The solutions were then kept in polypropelene bottles and kept at ∼4°C until analysis. Prior to their use, the bottles were cleaned repeatedly using distilled water and soaked for ∼72 hrs. The major ions, namely anions (Cl^−^, NO_3_^−^ and SO_4_^2−^) and cations (Na^+^, NH_4_^+^, K^+^, Ca^2+^ and Mg^2+^) were quantitatively determined by Ion Chromatograph (DIONEX-2000, USA) using analytical column IonPac ® AS15 with micro-membrane suppressor ASRS ultra II 2mm, 38 mM. KOH as eluent and triple distilled water as regenerator for anions. Similarly, the IonPac ® CS17 column with micro-membrane suppressor CSRS ultra II 2mm, 6 mM methansulfonic acid as eluent and triple distilled water as regenerator were used for cations. For calibration purpose, the standards were procured from Dionex for cations and anions. Detection limits of the ionic species, concentrations corresponding to three times the standard deviation of five replicate blank level measurements for Na^+^, K^+^, Ca^2+^, Mg^2+^, NH_4_^+^, Cl^−^, NO_3_^−^ and SO_4_^2−^ were 0.009, 0.0013, 0.003, 0.0015, 0.0024, 0.009, 0.005 and 0.008 µg m^−3^ respectively. The precision estimated from the standard deviation of repeat measurements of standard and samples was 2% for Na^+^, K^+^ and Ca^2+^; 3% for Mg^2+^; 5% for NH_4_^+^; 2% for Cl^−^, SO_4_^2−^ and 4% for NO_3_^−^. Trace gas SO_2_ was measured using an on-line SO_2_ analyzer (Horiba, APSA-360A) throughout all the sampling events at five minute interval.

## Results and Discussion

### 1. Seasonal variation of particulate matter

**Figure 5:**
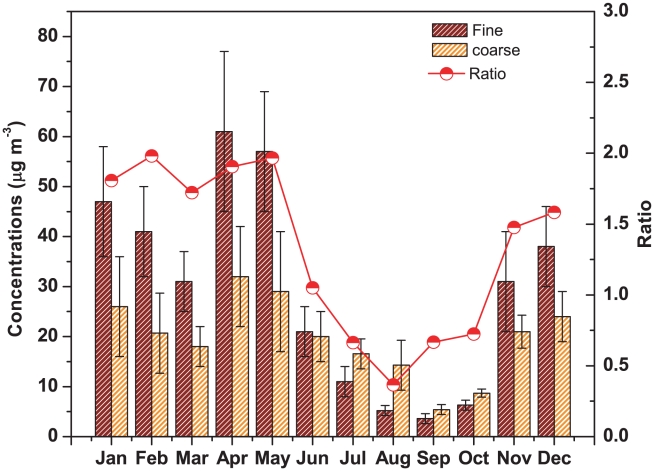
Seasonal variations of aerosol over Darjeeling.

The average concentration of fine mode aerosol in Darjeeling was found to be 29.5±20.8 µg m^−3^ varying between 3.6 µg m^−3^ and 61 µg m^−3^, whereas coarse mode aerosol ranged between 5.4 µg m^−3^ and 32 µg m^−3^ with an average of 19.6±11.1 µg m^−3^. The large variation in concentrations of both fine and coarse mode aerosol (Figure 5) during the entire study period could be due to the thermodynamic conditions in the planetary boundary layer (PBL), which either favor or adversely affect pollutants dispersion. Ambient weather conditions, such as air temperature, relative humidity and short wave radiation, could also influence the chemical reactions leading to secondary aerosol formation. Stable atmospheric conditions with a low mixing layer height may result in significantly enhanced particulate concentrations [16]. Aerosol shows higher concentrations during winter months and minimum concentrations during monsoon. In winter, very frequent and persistent thermal inversion and fog situations at ground level caused a considerable amount of aerosol to accumulate in the lower layers of the atmosphere [17]. Aerosol concentrations during winter were largely affected due to massive biomass burning over Darjeeling. The higher emission of K^+^, SO_4_^2−^ and carbonaceous species (not analyzed for the present study) could enhance the aerosol loading in the atmosphere during winter. The sharp fall in fine mode aerosol concentrations and high precipitation amount (1783 mm) during monsoon indicates the wash out effect of aerosol and its components. On the other hand, the coarse mode aerosol did not show a sharp decrease in concentrations during monsoon due to the contribution of sea salt aerosol (Na^+^, Cl^−^, Mg^2+^). Non-sea-sulphate and nitrate were also found to enhance the coarse mode aerosol concentrations during monsoon (discussed later in detail). The ratios of fine to coarse mode aerosol concentrations were found to widely vary between 0.36 (during August) and 4.4 (during January) with an average of 1.4±1.1. On an average, the average ratio of 1.72 was observed during dry seasons and 0.76 during monsoon. Thus, fine mode aerosol dominated over coarse mode aerosol during dry seasons whereas coarse mode aerosol dominated over fine mode aerosol during monsoon. A peak was observed in aerosol concentrations both in fine and coarse mode during two premonsoon months, April and May. This could be attributed to the long range transport of dust aerosol from arid regions of western India and even from Arabian deserts (discussed later in detail) during premonsoon. According to the elevated heat pump (EHP) mechanism proposed in [18] the dust aerosols mixed with carbonaceous aerosols primarily from Indo-Gangetic Plain (IGP) reaches the foothills of the Himalayas and are vertically advected to elevated altitudes. This causes significant loading of aerosol over the Himalayan region during premonsoon. The carbonaceous aerosol components and the trace elements (mainly the mineral dust component) data, though not analyzed for the present study, would provide better information on the high aerosol loading over Darjeeling during premonsoon.

### 2. Water-soluble ionic species in aerosol

#### 2.1. Comparison with other studies over Himalayan region and Indian subcontinent

Water-soluble inorganic ions (Na^+^, NH_4_^+^, K^+^, Ca^2+^, Mg^2+^, Cl^−^, NO_3_^−^, SO_4_^2−^) in fine and coarse mode aerosol over Darjeeling have been compared with the data reported in other high altitude stations in India and Nepal and other sites in Indian subcontinent.

For comparison with other high altitude stations, seven such stations (five in Nepal and two in India) have been chosen (Table 1). It is observed that the concentrations of sodium and chloride are several times higher than all the other hill stations indicating the strong influence of sea salt aerosol over Darjeeling. The most interesting feature is that in addition to the strong influence of sea salt aerosol, massive coal and biomass burning (domestic, industrial and from Darjeeling Himalayan Railways) throughout the year enhanced the concentration of non-sea-Cl^−^ in fine mode aerosol over Darjeeling. The concentrations of ammonium, nitrate and sulphate, the secondary anthropogenic ionic species in aerosol over Darjeeling, are 3–15 times higher than all the other hill stations except Nagarkot. The concentration of NH_4_^+^ in Nagarkot is higher than all the hill stations due to the close proximity of the sampling station to agricultural land and animal husbandry. The higher vehicular activities due to the high influx of tourists in Darjeeling could be the reason behind the higher loading of nitrate. Strong influence of massive biomass burning in Darjeeling was observed in the very high concentrations of non-sea-SO_4_^2−^ and non-sea-K^+^. When we compared the Ca^2+^ concentration, we noticed that our data do not differ significantly from the other high altitude stations. The concentration of Ca^2+^ varied between 0.12 µg m^−3^ and 0.75 µg m^−3^ in various stations in Himalayas (including Darjeeling), located between 1900 (Jiri, Nepal) and 5079 (NCO-P, Nepal) masl. Therefore, Ca^2+^ seems to be more homogeneously distributed vertically in Himalayan regions compared to other ionic species. The loading of dust aerosol enriched with Ca^2+^ in Himalayan region is mostly due to the long-range transport of dust aerosol originated in the western part of India (discussed later). However, the Ca^2+^ concentration over Mt. Abu in western India shows the highest concentration which is due to the widespread marble queries and stone-crushing mills in plain lands along the Aravali range in western India [19]. Mg^2+^ in the present study shows the highest concentration compared to the other sites. But if we look at the fine mode Mg^2+^ only, we find that our data do not significantly differ from the other sites (varying between 0.03 and 0.12 µg m^−3^) indicating homogeneous vertical distribution of Mg^2+^, like Ca^2+^, in Himalayan regions.

**Table 1:** Comparison of this study with other high altitude stations in India and Nepal.

	Darjeeling Northeastern Himalayas, India		Nagarkot, Nepal Himalayas	Langtang, Nepal Himalayas	Phortse, Nepal Himalayas	Jiri, Nepal Himalayas	Manora Peak, India	Khumbu valley, Nepal	Mt Abu, Western India
**References**	This study		[13]	[13]	[12]	[12]	[42]	[7]	[19]
**Altitude(masl)**	2194		2150	3920	4100	1900	1950	5079	1680
**Aerosol type**	Fine	Coarse	Fine	Fine	PM_10_	PM_10_	TSP	PM_10_	TSP
**Samples**	111	111	NA	NA	17	13	NA	13	25
**Na^+^**	0.66±0.43	2.20±2.00	0.20±0.16	0.12±0.18	0.03	0.08	NA	NA	0.28
**NH_4_^+^**	0.88±0.76	0.05±0.04	1.50±1.00	0.54±0.56	0.28	0.35	0.52	0.14	0.37
**K^+^**	1.20±0.80	0.31±0.21	0.62±0.58	0.20±0.26	0.15	0.25	0.23	0.02	0.20
**nsK^+^**	1.15±0.82	0.23±0.20	0.61	NA	NA	NA	NA	NA	NA
**Ca^2+^**	0.13±0.01	0.40±0.20	0.31±0.29	0.66±0.89	0.12	0.56	0.75	0.10	1.70
**nsCa^2+^**	0.11±0.10	0.38±0.21	NA	NA	NA	NA	NA	NA	NA
**Mg^2+^**	0.12±0.06	0.31±0.17	0.04±0.03	0.05±0.05	0.03	0.08	NA	NA	0.09
**nsMg^2+^**	0.04±0.03	0.14±0.18	NA	NA	NA	NA	NA	NA	NA
**Cl^−^**	1.21±1.00	2.35±1.50	0.05±0.05	0.06±0.10	0.01	0.01	NA	NA	0.31
**nsCl^−^**	0.44±0.41	−0.18±1.10	NA	NA	NA	NA	NA	NA	NA
**NO^3−^**	3.31±2.30	0.95±0.20	1.20±1.80	0.78±0.10	0.70	0.48	0.50	0.08	0.74
**SO_4_^2−^**	3.80±2.90	2.50±2.10	3.80±1.00	1.41±1.30	0.98	0.41	2.60	0.22	2.71
**nsSO_4_^2−^**	3.64±2.80	1.92±1.84	NA	NA	NA	NA	NA	NA	NA

NA: Not Available; Concentrations of ionic species are given in µg m^−3^.

The comparison of the data of aerosol ionic species of the present study with four urban sites (including a mega city, Mumbai) in India is shown in Table 2. It is observed from the table that, Na^+^ and Cl^−^ are higher in concentrations than Pune and Ahmedabad and comparable to Agra, but the most interesting feature is that the concentrations of Na^+^ and Cl^−^ in Darjeeling are found to be higher than at Mumbai, a coastal city in western India. At this stage, we could not find a fool-proof explanation for this most interesting observation; further, long-term study is required to understand this phenomenon. NH_4_^+^ and NO_3_^−^ in Darjeeling are found to show lower concentrations than at Mumbai, Pune or Agra. K^+^ and SO_4_^2−^ show higher concentrations in Darjeeling than at Pune and Ahmedabad whereas we observed a similarity in SO_4_^2−^ concentrations between Darjeeling and Mumbai. The concentrations of Ca^2+^ over all the urban sites are found to be higher than that over Darjeeling. The very high Ca^2+^ concentration over Ahmedabad is due to its close proximity to Thar deserts. On the other hand, the concentration of Mg^2+^ in Darjeeling is found to be higher than Pune and Ahmedabad and lower than Mumbai and Agra.

**Table 2:** Comparison of this study with other urban sites in India.

	Darjeeling, Northeastern Himalayas, India		Mumbai, India	Pune, India	Agra, India	Ahmedabad, India
**References**	This study		[43]	[9]	[44]	[19]
**Altitude(masl)**	2194					
**Aerosol type**	Fine	Coarse	PM_10_	PM_10_	TSP	TSP
**Samples**	111	111	NA	NA	NA	25
**Na^+^**	0.66±0.43	2.20±2.00	2.20	0.48	2.97	0.81
**NH_4_^+^**	0.88±0.76	0.05±0.04	NA	2.14	6.52	0.48
**K^+^**	1.20±0.80	0.31±0.20	8.90	0.43	2.50	0.76
**nsK^+^**	1.15±0.82	0.23±0.20	NA	NA	NA	0.73
**Ca^2+^**	0.13±0.01	0.40±0.20	6.20	2.50	3.02	2.96
**nsCa^2+^**	0.11±0.10	0.38±0.20	NA	NA	NA	2.93
**Mg^2+^**	0.12±0.06	0.31±0.17	2.20	0.23	1.24	0.25
**nsMg^2+^**	0.04±0.03	0.14±0.18	NA	NA	NA	0.15
**Cl^−^**	1.21±1.00	2.35±1.50	2.60	1.80	6.78	0.99
**nsCl^−^**	0.44±0.41	−0.18±1.10	NA	NA	NA	NA
**NO^3−^**	3.31±2.30	0.95±0.20	6.00	2.91	8.37	2.07
**SO_4_^2−^**	3.80±2.90	2.50±2.10	6.20	2.98	14.7	4.57
**nsSO_4_^2−^**	3.64±2.80	1.92±1.84	NA	NA	NA	4.36

NA: Not available; Concentrations of ionic species are given in µg m^−3^.

Conclusively, we observed that Darjeeling in northeastern Himalayas is strongly influenced by the sea salt and anthropogenic aerosol which are higher than all the high altitude stations in Indian and Nepal Himalayan region and even higher than most of the urban regions in India, compared with the present study. On the other hand, dust aerosol is found to be homogeneously distributed vertically between all the Himalayan regions but is significantly lower than urban regions in India.

#### 2.2. Secondary ionic species in fine and coarse mode aerosol

NH_4_^+^, NO_3_^−^ and SO_4_^2−^, the secondary components of aerosol, constituted 67.8±5.9% fine mode and 36.4±10.07% coarse mode aerosol in Darjeeling. The average concentrations of NH_4_^+^, NO_3_^−^ and SO_4_^2−^ in fine and coarse mode aerosol were 0.88±0.76 µg m^−3^, 3.31±2.25 µg m^−3^, 3.8±2.9 µg m^−3^ and 0.05±0.04 µg m^−3^, 0.95±0.17 µgm^−3^, 2.5±2.1 µg m^−3^, respectively.

**Figure 6:**
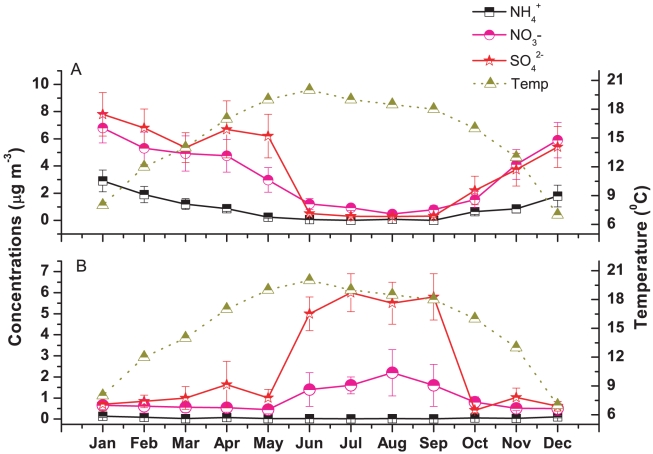
Seasonal variations of secondary ionic species and temperature over Darjeeling.

NH_4_^+^ and NO_3_^−^ in fine mode aerosol showed higher enrichment (∼15 times and 4 times, respectively) compared to coarse mode aerosol. This is due to the fact that gas to particle conversion of gas phase HNO_3_ and NH_3_ to particulate NO_3_^−^ and NH_4_^+^ are more feasible in nucleation (<0.1µm) and accumulation mode (>0.1 µm <2.5 µm) particles [20]. NH_4_^+^ and NO_3_^−^ show good correlation (with correlation coefficient, R^2^ = 0.74 and number of samples, n = 81) (Table 3) between each other in fine mode aerosol during dry seasons. Figure 6 shows the month wise variations in secondary species concentrations along with ambient temperature both in fine and coarse mode aerosol. The variation of fine mode NH_4_^+^ and NO_3_^−^ are similar in nature showing a gradual decrease in concentrations from the month of January with a minimum during monsoon and a gradual increase till December which is exactly opposite in nature with respect to the ambient temperature variations. The higher NH_4_^+^ concentrations during winter is under the influence of NE wind transporting the large-scale pollutants whereas the lower concentration of NH_4_^+^ in monsoon suggests the dominant occurrence of NH_4_^+^ in gas phase [19].

**Table 3:** Inter-ionic correlations in fine and coarse mode aerosols over Darjeeling during dry seasons (n = 81).

	Na^+^	NH_4_^+^	K^+^	nsK^+^	Ca^2+^	nsCa^2+^	Mg^2+^	nsMg^2+^	Cl^−^	NO_3_^−^	SO_4_^2−^	nsSO_4_^2−^
**Na^+^**		F = 0.35C = 0.14	F = 0.24C = 0.21	F = 0.16C = 0.13	F = 0.26C = 0.23	F = 0.18C = 0.39	F = 0.13C = 0.19	F = 0.11C = 0.29	F = 0.38C = 0.44	F = 0.17C = 0.11	F = 0.19C = 0.37	F = 0.14C = 0.26
**NH_4_^+^**			F = 0.12C = 0.16	F = 0.19C = 0.31	F = 0.11C = 0.10	F = 0.17C = 0.21	F = 0.22C = 0.20	F = 0.19C = 0.14	F = 0.23C = 0.10	F = 0.74C = 0.51	F = 0.44C = 0.42	F = 0.65C = 0.44
**K^+^**					F = 0.18C = 0.38	F = 0.21C = 0.34	F = 0.16C = 0.18	F = 0.14C = 0.34	F = 0.26C = 0.37	F = 0.11C = 0.15	F = 0.19C = 0.27	F = 0.29C = 0.27
**nsK^+^**					F = 0.29C = 0.31	F = 0.31C = 0.21	F = 0.11C = 0.20	F = 0.13C = 0.42	F = 0.82C = 0.57	F = 0.19C = 0.27	F = 0.39C = 0.17	F = 0.78C = 0.37
**Ca^+2^**							F = 0.55C = 0.62	F = 0.48C = 0.42	F = 0.19C = 0.22	F = 0.19C = 0.27	F = 0.22C = 0.31	F = 0.11C = 0.25
**nsCa^2+^**							F = 0.39C = 0.54	F = 0.64C = 0.74	F = 0.10C = 0.17	F = 0.10C = 0.22	F = 0.31C = 0.37	F = 0.20C = 0.12
**Mg^+2^**									F = 0.23C = 0.31	F = 0.10C = 0.17	F = 0.32C = 0.31	F = 0.25C = 0.27
**nsMg^2+^**									F = 0.30C = 0.12	F = 0.16C = 0.17	F = 0.15C = 0.17	F = 0.11C = 0.26
**Cl^−^**										F = 0.19C = 0.15	F = 0.29C = 0.48	F = 0.78C = 0.37
**NO_3_^−^**											F = 0.58C = 0.33	F = 0.55C = 0.41
**SO_4_^−2^**												
**nsSO_4_^2−^**												

The higher concentration of particulate ammonium and nitrate in winter was due to the shifting from the gas phase of nitric acid to the particulate phase of nitrate at lower temperature. The formation of particulate NH_4_NO_3_ is given by the following equilibrium
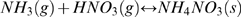
The equilibrium shifts towards the left side at higher temperature as NH_4_NO_3_ volatilizes when temperature increases [21]. We observed a gradual decrease in fine mode ammonium and nitrate concentrations from January as temperature increases. Also, when there is significant production of sulphate by reaction between OH radical and SO_2_, then sulphate acts as a sink for ammonia. Once ammonia becomes ammonium bisulphate or ammonium sulphate, ammonium becomes unavailable for nitrate [22]. We observed the high sulphate production in fine mode aerosol through SO_2_ oxidation by OH radical (discussed in section 4.2) during premonsoon. While fine mode nitrate shows minimum concentrations during monsoon due to the wash out effect or below cloud scavenging, the coarse mode NO_3_^−^ shows higher concentrations during monsoon compared to the dry seasons. The presence of sea salt aerosol may favor the formation of coarse mode NO_3_^−^ as NaNO_3_ by up taking of nitrogen oxides and HNO_3_ on the surface of sea salt aerosol [23]. Many investigators have observed that the depletion of Cl^−^ from sea salt aerosol and the simultaneous occurrence is particularly pronounced in coarse particle, typical of sea salt aerosol [23]. The role of nitrate in chloride depletion from sea salt aerosol has been discussed in detail in section 4.2.

**Figure 7:**
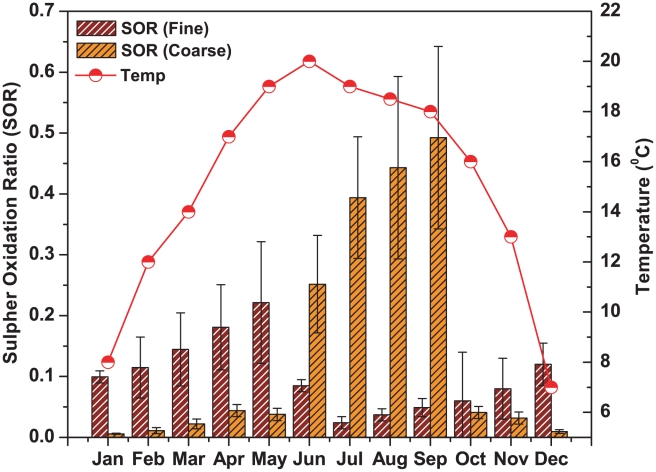
Different mechanisms of sulphate production over Darjeeling in different seasons.

The sulfur oxidation ratio defined as 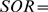 
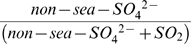
 was used as an indicator of the secondary transformation process and the formation route and source of sulphate in the atmosphere. Figure 7 shows the month wise variation of SOR with temperature for both fine and coarse mode aerosol. The ratio varied between 0.03 and 0.22 with an average of 0.11±0.07 in fine mode aerosol and between 0.006 and 0.49 with an average of 0.15±0.14 in coarse mode aerosol. SORs in fine mode aerosol were found to increase gradually with the temperature and higher values were observed during premonsoon. When the ratio value is greater than 0.10, then there would be the occurrence of the photochemical oxidation of SO_2_ in the atmosphere [24]. This indicates that gas phase photochemical oxidation of SO_2_ followed by the condensation and absorption into the particle phase was the most important pathway for the production of fine mode SO_4_^2−^ in the atmosphere during premonsoon (SOR>0.1). The gas phase oxidation of SO_2_ to SO_4_^2−^ by OH radical is a strong function of temperature [25]. The comparatively lower SOR during the winter months for both the fine and coarse mode aerosols indicate some aqueous phase transformation processes such as metal catalyzed oxidation of SO_2_, aqueous phase H_2_O_2_/O_3_ oxidation of SO_2_ etc. Similar observation was also made in [26]. On the other hand, the very high SOR in coarse mode aerosol during monsoon could be due to the absorption of SO_2_ by the soil dust particles at higher relative humidity during monsoon to form coarse mode sulphate of crustal origin [19], [27]. Table 4 shows strong correlations (with correlation coefficient, R^2^ = 0.68 and number of samples, n = 30) non-sea-SO_4_^2−^ and non-sea-Ca^2+^ and between non-sea-SO_4_^2−^ and non-sea-Mg^2+^ (R^2^ = 0.68, n = 30) in coarse mode aerosol during monsoon.

**Table 4:** Inter-ionic correlations in fine and coarse mode aerosols over Darjeeling during monsoon (n = 30).

	Na^+^	NH_4_^+^	K^+^	nsK^+^	Ca^2+^	nsCa^2+^	Mg^2+^	nsMg^2+^	Cl^−^	NO_3_^−^	SO_4_^2−^	nsSO_4_^2−^
**Na^+^**		F = 0.15C = 0.14	F = 0.14C = 0.11	F = 0.17C = 0.22	F = 0.10C = 0.11	F = 0.19C = 0.23	F = 0.15C = 0.23	F = 0.17C = 0.31	F = 0.68C = 0.89	F = 0.12C = 0.83	F = 0.29C = 0.39	F = 0.78C = 0.21
**NH_4_^+^**			F = 0.16C = 0.19	F = 0.21C = 0.41	F = 0.23C = 0.31	F = 0.11C = 0.22	F = 0.09C = 0.13	F = 0.08C = 0.03	F = 0.15C = 0.10	F = 0.14C = 0.31	F = 0.11C = 0.12	F = 0.10C = 0.14
**K^+^**					F = 0.11C = 0.18	F = 0.16C = 0.11	F = 0.05C = 0.11	F = 0.03C = 0.02	F = 0.13C = 0.09	F = 0.11C = 0.19	F = 0.23C = 0.16	F = 0.09C = 0.07
**nsK^+^**					F = 0.39C = 0.13	F = 0.10C = 0.21	F = 0.11C = 0.08	F = 0.10C = 0.06	F = 0.08C = 0.02	F = 0.19C = 0.31	F = 0.29C = 0.11	F = 0.33C = 0.41
**Ca^+2^**							F = 0.20C = 0.32	F = 0.11C = 0.12	F = 0.09C = 0.06	F = 0.10C = 0.25	F = 0.21C = 0.30	F = 0.14C = 0.15
**nsCa^2+^**							F = 0.19C = 0.04	F = 0.16C = 0.13	F = 0.15C = 0.12	F = 0.13C = 0.21	F = 0.08C = 0.06	F = 0.13C = 0.72
**Mg^+2^**									F = 0.33C = 0.68	F = 0.13C = 0.03	F = 0.12C = 0.06	F = 0.07C = 0.06
**nsMg^2+^**									F = 0.11C = 0.03	F = 0.16C = 0.02	F = 0.04C = 0.10	F = 0.09C = 0.68
**Cl^−^**										F = 0.21C = 0.15	F = 0.31C = 0.58	F = 0.09C = 0.07
**NO_3_^−^**											F = 0.15C = 0.15	F = 0.16C = 0.13
**SO_4_^−2^**												
**nsSO_4_^2−^**												

#### 2.3. Primary ionic species in fine and coarse mode aerosol

**Figure 8:**
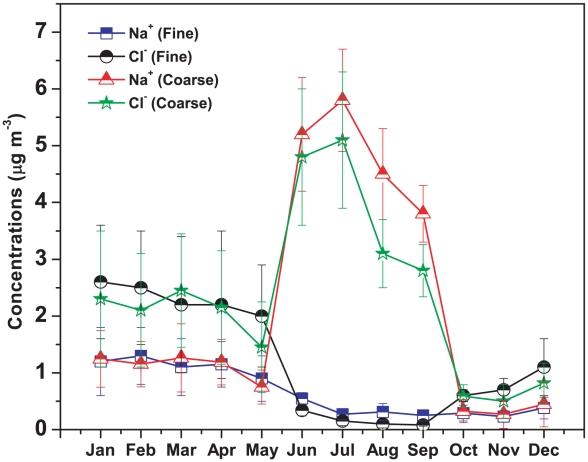
Seasonal variations of primary ionic species over Darjeeling.

Na^+^ and Cl^−^ constituted 16.6±5.46% fine mode and 46.2±11.14% coarse mode aerosol. The average concentrations of Na^+^ and Cl^−^ in fine and coarse mode aerosol were found to be 0.66±0.43 µg m^−3^, 1.21±1.00 µg m^−3^ and 2.16±2.01 µg m^−3^, 2.35±1.5 µg m^−3^ respectively. The seasonal distribution of Na^+^ and Cl^−^ in coarse mode aerosol is similar in nature with the higher concentrations during monsoon. But the concentration of fine mode Cl^−^ was found to be higher during the winter months and minimum during monsoon (Figure 8).

During monsoon, the correlation between coarse mode Na^+^ and Cl^−^ was found to be very strong (R^2^ = 0.89, n = 30) (Table 4). The month wise variation of Na^+^ and Cl^−^ (Figure 8) for both fine and coarse mode aerosol show that during monsoon, coarse mode Na^+^ and Cl^−^ show higher concentrations whereas fine mode Na^+^ and Cl^−^ show minimum concentrations. This indicates that both the coarse mode Na^+^ and Cl^−^ have a common source *i.e.* sea salt particle, which could be transported by the southwest monsoon. Monsoon air masses reaching Darjeeling originate in the Bay of Bengal and incorporate a large amount of Na^+^ and Cl^−^. The inter-tropical convergence zone (ITCZ) is aligned at a rather northerly position during the monsoon season. The air masses are vertically raised by convective motion and transported horizontally by the upper air southerly monsoon flow to the Himalayas [12]. The higher concentrations of fine and coarse mode Cl^−^ compared to Na^+^ during the dry seasons (Jan–May, Oct–Dec) is believed to be associated with biomass and coal burning (domestic and railway). We also observed strong correlations between non-sea-K^+^ and Cl^−^ (R^2^ = 0.82, n = 81) and between non-sea-SO_4_^2−^ and Cl^−^ (R^2^ = 0.78, n = 81) during dry seasons (Table 3) indicating the common biomass-burning source.

**Figure 9:**
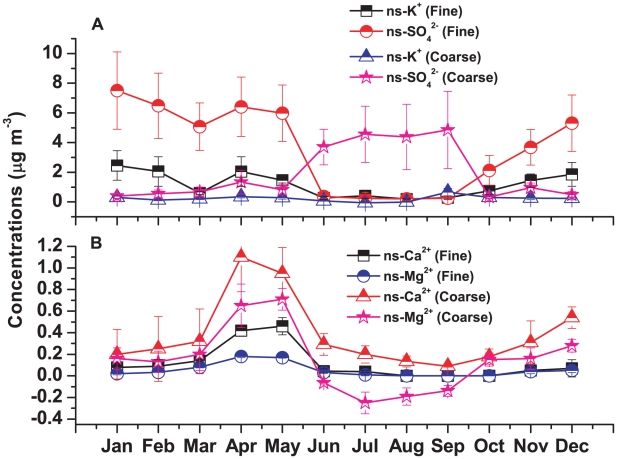
Seasonal variations of non-sea (ns) aerosol ionic species. A) Anthropogenic aerosols (biomass burning) and B) Natural aerosols (dust).

The average concentrations of K^+^ in fine and coarse mode aerosol were 1.17±0.83 µg m^−3^ and 0.31±0.17 µg m^−3^, respectively. Biomass burning could have the highest abundance of fine mode K^+^ of all source emissions. The fine mode K^+^ could be released in the atmosphere by the burning of vegetative scrap [28]. The average concentration of fine mode non-sea-K^+^ during winter was much higher (2.12±0.4 µg m^−3^) than premonsoon (1.37±0.8 µg m^−3^), monsoon (1.15±0.7 µg m^−3^) and post-monsoon (1.08±0.62 µg m^−3^) which was due to the massive biomass burning around Darjeeling especially during night times in winter. Figure 9(A) shows the month wise variations of fine mode non-sea-K^+^ which is similar to fine mode non-sea-SO_4_^2−^ variations. A strong correlation (R^2^ = 0.78, n = 81) between non-sea-K^+^ and non-sea-SO_4_^2−^ was also observed in fine mode aerosol during dry seasons (Table 3) indicating their common source of biomass burning.

The average concentrations of Ca^2+^ and Mg^2+^ in fine mode aerosol were 0.13±0.1 µg m^−3^ and 0.12±0.09 µg m^−3^ respectively and in coarse mode aerosol were 0.39±0.19 µg m^−3^ and 0.31±0.17 µg m^−3^ respectively. Figure 9(B) shows the monthly variations of non-sea-Ca^2+^ and non-sea-Mg^2+^ both in fine and coarse mode aerosol. Strong correlations were observed between non-sea-Ca^2+^ and non-sea-Mg^2+^ (Table 3) during dry seasons indicating their enrichment in aerosol mainly as dust aerosol. Non-sea-Ca^2+^ and non-sea-Mg^2+^ both show peaks during pre-monsoon both in fine and coarse mode aerosol. The higher concentrations of those mineral components during pre-monsoon could be related to the long-range transport of dust aerosol. The dust aerosols driven by the premonsoon westerlies are vertically advected to elevated altitudes (∼5 km) against the foothills of the Himalayas due to the enhanced convection and steep pressure gradient across the Himalayan-Gangetic region [29]. According to [29], the dust-rich aerosols from the Indo-Gangetic plain can “climb” the slope of the Himalayas in the premonsoon season. Similar concentrations of Ca^2+^ at two different altitudes, 800 and 3920 masl at two stations in Nepal Himalayan region during premonsoon was also observed showing the long-range transport from southwestern Asia and northern Africa [13]. Very high concentrations of coarse mode Ca^2+^ over a station near Thar deserts was also observed [30], [31].

**Figure 10:**
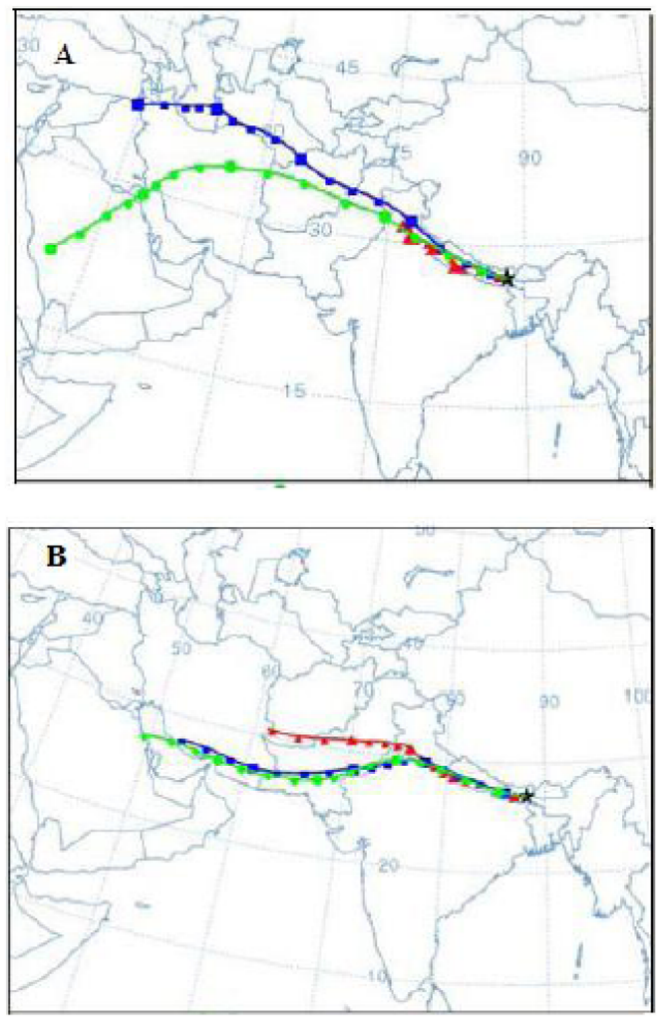
Long range transport of dust aerosol. A) From Thar deserts and B) From Arabian deserts.

Using HYSPLIT_4 (Hybrid Single Particle Lagrangian Integrated Trajectory, www.arl.noaa.gov/ready/hysplit4.html) model developed by NOAA/ARL, back trajectories were computed for all sampling events at 0500 UTC. Two distinct source regions are identified, arid and semi-arid regions of western India including Thar deserts (45%) and upwind regions of Arabian deserts (32%), which are shown in figure 10A and 10B as representative figures. The percentage distributions of source regions (45% from Thar deserts and 32% from Arabian deserts) were determined based on the ratio of the number of events of the respective regions (using HYSPLIT) to the total number of sampling events. The loading of Ca^2+^ and Mg^2+^ over Darjeeling from Thar deserts was found to be higher than the Arabian deserts. The correlation of Ca^2+^ and Mg^2+^ with trace metals like Fe, Al, Si during premonsoon would be useful to put the evidence towards the transport of dust aerosol from distant regions. However the studies on the effect of dust aerosol on the total aerosol loading over Himalayan region and its effect on the optical properties of aerosol during premonsoon are under development.

The higher concentrations of Ca^2+^ over Ahmedabad (located near Thar desert in western India) compared to Darjeeling and other Himalayan regions (Table 1 and 2) indicate that the dust aerosol reach Himalayas after significant dilution. With the onset of rainy season (the Arabian Sea and Bay of Bengal branches of the southwest summer monsoon), the heavy dust loading significantly diminishes due to aerosol washout from the atmosphere. Non-sea-Ca^2+^ and non-sea-Mg^2+^ both show minimum concentrations indicating the below cloud scavenging of dust aerosol due to heavy rain during monsoon (1783 mm). However, the negative concentration of coarse mode non-sea-Mg^2+^ during monsoon indicates that the entire coarse mode Mg^2+^ was from the marine source during monsoon.

#### 2.4. Chloride depletion: interaction between marine and urban aerosol

**Figure 11:**
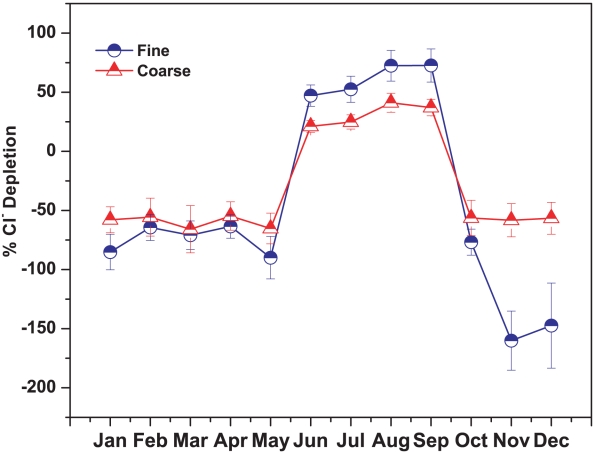
Seasonal variation of chloride depletion from sea-salt aerosol.

Chloride depletion results when the acidic species, mainly nitrate, sulphate and some organic acids react with sea-salt particles and replace Cl^−^ in the form of HCl gas. NOx transforms into gaseous nitrous and nitric acid, which react with NaCl in sea-salt aerosol to form NaNO_3_ and HCl. SO_2_ oxidation and to a lesser extent H_2_SO_4_ vapor condensation on sea salt aerosols can also lead to chloride depletion. The reaction pathways are given as follows [32]:
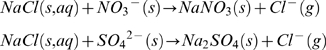
Those reactions given above occur especially when polluted urban aerosols and maritime aerosols are mixed with each other. Thus the extent of chloride depletion is an indication of the interaction between marine and urban aerosol as well as important in estimating the amount of nitrate and sulphate formed on sea-salt particles. Figure 11 shows the monthly variations of chloride depletion and it is clearly observed from the figure that only monsoon shows chloride depletion both in fine and coarse mode aerosol. The percentage of Cl^−^ depletion (Cl^−^_dep_) can be calculated as follows [32]:
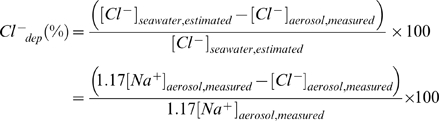
Here, the source of measured Na^+^ concentration in aerosol is assumed to be sea water only. In this study, the average depletion of Cl^−^ in fine mode aerosol (61.2±13.3%) during monsoon was higher than coarse mode aerosol (31.02±9.6%). It was suggested that the surface reaction mechanism is the principal explanation for higher depletion of smaller particles [33], [34]. The dynamics of the chloride depletion reactions favor smaller particles because of their larger surface area distribution and longer atmospheric residence time. [32] observed that the Cl^−^ depletion decreases from 98% to 10% as particle size increases from 1.8 µm to 18 µm. [33] reported a reduction of chloride depletion of 90–100% at particle size of 1–2 µm to less than 40% at particle size of 8–15 µm for sea-salt particles in Finland.

**Figure 12:**
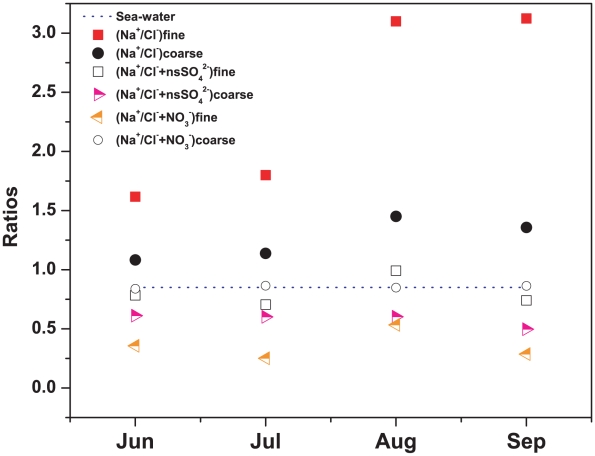
Modified ratios of sodium to chloride indicating contribution of nitrate and non-sea (ns) sulphate to chloride depletion.

An attempt was made to estimate the contribution of nitrate and non-sea-sulphate to the chloride depletion in fine and coarse mode aerosol. A ratio of the measured Na^+^ concentrations in aerosol to the estimated original Cl^−^ concentration in sea salt was used to determine the contribution of nitrate and non-sea-sulphate to chloride depletion. According to [33] the original chloride concentrations in sea salt can be determined as 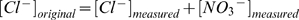, assuming that chloride depletion results from nitrate formation process and the amount of measured nitrate is equal to the amount of lost chloride or 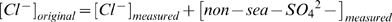, assuming that chloride depletion results from non-sea-sulphate formation process and the amount of measured non-sea-sulphate is equal to the amount of lost chloride or 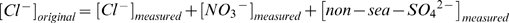, assuming both nitrate and non-sea-sulphate are responsible for the depletion of chloride. Figure 12 shows the scatter plot of the ratios during monsoon for fine and coarse mode aerosol along with the ratio of measured [Na^+^] to measured [Cl^−^] in aerosol. The ratio, [Na^+^]/[Cl^−^] in unreacted original sea water is 0.85.

Naturally the ratio, [Na^+^]/[Cl^−^], in fine and coarse mode aerosol were higher than 0.85 because of chloride depletion. Interestingly, the ratios [Na^+^]/([Cl^−^]+[NO_3_^−^]) for coarse mode aerosol were found to be close to 0.85. This indicates that the measured coarse mode nitrate during monsoon was involved in chloride depletion. A strong correlation between coarse mode Na^+^ and coarse mode NO_3_^−^ during monsoon (R^2^ = 0.83, n = 30) (Table 4) added further evidence to the above fact. On the other hand, the ratios [Na^+^]/([Cl^−^]+[NO_3_^−^]) for fine mode aerosol were quite lower than 0.85 indicating the overestimation of nitrate, *i.e.* the measured nitrate was not totally the nitrate from sea-salt but also from some other sources. The ratios, [Na^+^]/([Cl^−^]+[non-sea-SO_4_^2−^]) in fine mode aerosol were close to 0.85. This indicates that the measured fine mode non-sea-sulphate during monsoon was totally involved in chloride depletion. We observed a strong correlation between Na^+^ and fine mode non-sea-SO_4_^2−^ (R^2^ = 0.78, n = 30) (Table 4) during monsoon. Thus, we observed that non-sea-sulphate depleted chloride from fine mode sea salt aerosol whereas nitrate depleted chloride from coarse mode sea salt aerosol.

The chloride depletion from sea salt aerosol *i.e.* the interaction between marine (natural) and urban (anthropogenic) aerosol has major implications not only in changing the chemical composition of aerosol in different size modes but also it governs the rate of dry atmospheric removal of the species involved in chloride depletion. For example, here in this study the chloride depletion from coarse mode sea salt aerosol was found to govern the dry atmospheric removal of nitrate from the atmosphere. The dry depositional flux of nitrate in fine and coarse mode aerosol was calculated as follows:
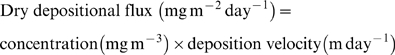
Here, deposition velocity of nitrate in fine mode aerosol was considered to be 0.1 cm s^−1^ and that in coarse mode was considered to be 1 cm s^−1^ [35]. It is relevant to state that these deposition velocities can be in error up to a factor of 3 [36]. The nitrate was found to be enriched mainly in fine mode aerosol as NH_4_NO_3_ during winter with the average deposition flux of 518±65 mg m^−2^ day^−1^. On the other hand, coarse mode nitrate was found to be enriched as NaNO_3_ after depleting chloride from sea salt aerosol during monsoon with a higher deposition flux of 1468±300 mg m^−2^ day^−1^. Thus we observed that the interaction between natural marine aerosol and anthropogenic urban aerosol enhanced the rate of dry atmospheric removal of nitrate.

### 3. Acidity of aerosol: ionic balance and ionic ratios

Strong acidity in terms of nmol H^+^ per m^3^ of air is a parameter that characterizes the absolute acidity of atmospheric aerosols [37]. This is obtained from the total H^+^ derived from the aqueous extract of atmospheric aerosols and is estimated using an ionic balance of the inorganic ionic species. On the other hand, free acidity (pH) characterizes the relative acidity of atmospheric aerosols and depends on the water content and composition of aerosol and relative humidity [36]. Here in this study we have given more emphasis on ionic balance in explaining the aerosol acidity. Although strong acidity is due to the strong acidic components in aerosols like nitrate and sulphate, we have estimated the acidity (total H^+^ in nmol m^−3^) using all the ionic species according to [38].
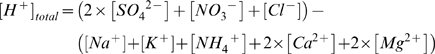

**Figure 13:**
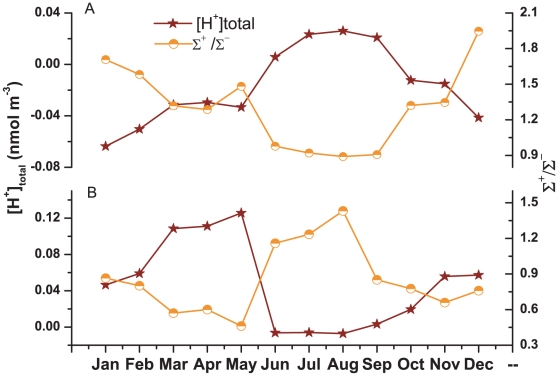
Seasonal variation of acidity of aerosol and ionic ratios.

Figure 13 shows the month wise variations of [H^+^]_total_ and the equivalent ratio of the sum of cations to the sum of anions (Σ^+^/Σ^−^) in fine and coarse mode aerosol. It was observed that fine mode aerosol acidity ([H^+^]_total_) was much higher during dry seasons (73 nmol m^−3^) compared to monsoon (−4.1 nmol m^−3^) whereas coarse mode aerosol acidity was higher during monsoon (19 nmol m^−3^) compared to dry seasons (−34.6 nmol m^−3^). The ratio, Σ^+^/Σ^−^, ranged between 0.46 to 1.43 (Average: 0.84, Standard deviation: 0.29) in fine mode aerosol and between 0.9 to 1.95 (Average: 1.31, Standard deviation: 0.34) in coarse mode aerosol. The cation deficiency in fine mode aerosol could be attributed to H^+^ whereas anion deficiency in coarse mode aerosol could be attributed to unanalyzed organic acid ions, carbonate, bicarbonate etc. The higher concentrations of nitrate, non-sea-sulphate and chloride during dry seasons increased [H^+^]_total_ in fine mode aerosol whereas the lower concentrations of non-sea-magnesium and non-sea-calcium along with higher concentration of nitrate during monsoon increased [H^+^]_total_ in coarse mode aerosol. Thus, the acidity of fine mode aerosol was mainly controlled by the species originated from fossil fuel and biomass burning whereas the acidity of coarse mode aerosol was mainly controlled by dust particles.

### 4. Sources of aerosol

#### 4.1. Source apportionment by Principal Component Analysis

It is very important, and difficult as well, to identify the exact sources of the aerosol components in Darjeeling, the area under concern at the northeastern Himalayas, where composite anthropogenic activities including biomass burning and vehicular emission play a central role in loading of air pollutants in the atmosphere. Principal component analysis (PCA), a multivariate analysis technique [39] was used to identify possible sources of aerosols in Darjeeling. Each principal component (PC) shows correlation of each variable as loadings (loading greater than 0.5 was considered to be statistically significant in this study). Since higher loading of particular variable in a PC can help in identifying the possible sources [40], the number of PCs selected (sources identified) should represent the sources that are relevant in the receptor domain. PCA was performed using the statistical software, SPSS (Statistical Package for the Social Sciences) [41] of version 16.0.2 using the data sets over the entire study period (n = 66).

**Figure 14:**
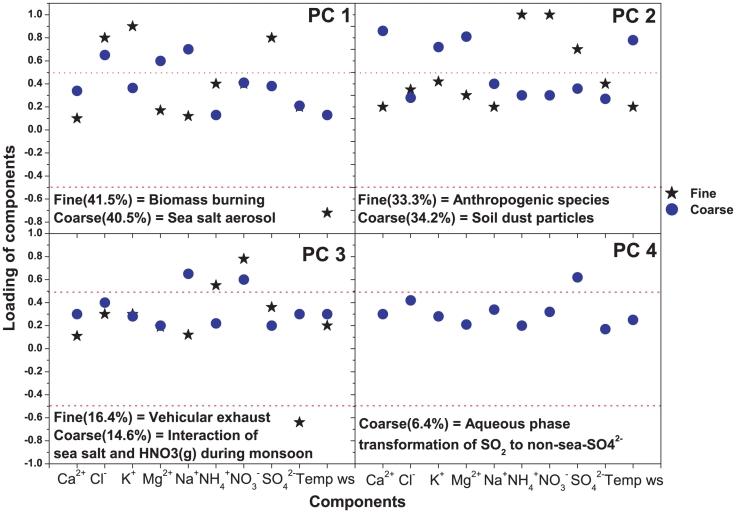
Aerosol source apportionment by Principal Component Analysis.

For fine mode aerosol, three PCs were extracted (Figure 14). The first PC (PC1) shows the heavy loading of K^+^, Cl^−^ and SO_4_^2−^ with wind speed having 41.5% variance of the data set. These species in PC1 correspond to the massive biomass burning throughout the year mainly during winter in Darjeeling. The coal engine which is used for the Darjeeling Himalayan Railways is also a major source of these species in the atmosphere. Thus, PC1 indicates the loading of K^+^, Cl^−^ and SO_4_^2−^ as non-sea-K^+^, non-sea-Cl^−^ and non-sea-SO_4_^2−^ respectively. The negative loading of wind speed in this PC indicates the dispersion of these fine mode species favored by higher wind speed. The second PC (PC2) shows the heavy loading of NH_4_^+^, NO_3_^−^, SO_4_^2−^ with 33.3% of the data variance. This PC is associated with the formation of secondary anthropogenic particles in the atmosphere. Different agricultural activities and usage of different ammoniated fertilizers in several tea gardens and also in tea processing plants, animal manure and human activities are the major sources for the emission of NH_3_ and/or NH_4_^+^ in the atmosphere. The third PC (PC3) is moderately loaded with NH_4_^+^ and highly loaded with NO_3_^−^ along with temperature with 16.4% of the data variance. This indicates the emission of NOx from vehicular exhaust and its subsequent transformation to particulate nitrate mostly as ammonium nitrate. The negative loading of temperature in PC3 clearly indicates the inverse relation of formation of particulate nitrate with temperature as discussed earlier.

For coarse mode aerosol, four PCs were extracted (Figure 14). The first PC (PC1) is highly loaded with the Na^+^, Cl^−^ and Mg^2+^ having 40.5% data variance. This indicates the loading of sea salt aerosol. The second PC (PC2) shows the heavy loading of Ca^2+^, Mg^2+^ along with wind speed with the moderate loading of K^+^. This explains 34.2% of the data variance and indicates the enrichment of calcium, magnesium and a fraction of potassium as non-sea-calcium, non-sea-magnesium and non-sea-potassium from soil dust particles. The loading of wind speed in this PC indicates the resuspension of soil dust in the atmosphere favored by the higher wind speed. The third PC (PC3) shows the loading of Na^+^ and NO_3_^−^ with the data variance of 14.6%. This indicates the interaction of sea salt aerosol (NaCl) with nitrate (particulate or gaseous) and the production of coarse mode nitrate through the chloride displacement reactions discussed earlier. The fourth PC (PC4) shows the higher loading and association of SO_4_^2−^ only with 6.4% of data variance. The loading of sulphate singly in PC4 indicates the enrichment of non-sea-sulphate in coarse mode which is due to the aqueous phase transformation of SO_2_ to sulphate in high relative humidity during monsoon. The higher concentration of non-sea-sulphate during monsoon in coarse mode aerosol has already been discussed.

#### 4.2. Source identification by air mass parcels using HYSPLIT back trajectory

In order to find out the possibility of atmospheric transport of different species from outside of Darjeeling, backward trajectories for air parcels arriving at the sampling site during the sampling period were calculated with the HYSPLIT_4 model. Back trajectories were computed for all sampling events at 0500 UTC. The Final Run (FNL) meteorological data was used for the trajectory calculation.

Different air masses travel through different regions and bring different chemical components with the aerosol, thus the distribution of chemical components among different air masses could shed some light on their possible sources. Based on the transport pathways of air masses, three typical air mass trajectories, representing local, continental (other than local), and marine were found in Darjeeling and have been shown in Figure 14. Marine air parcels are mainly from the South East (Bay of Bengal) and South West (Arabian Sea) and could bring a large amount of marine aerosol. Continental air masses are mainly from North West and could bring several crustal species from inland emissions. Table 5 presents the distributions of mass concentrations of major ionic species along with [H^+^]_total_ (nmol m^−3^) and Σ^+^/Σ^−^ ratio both in fine and coarse mode aerosol for three types of air masses.

**Table 5:** Concentrations of water soluble ionic species in fine and coarse mode aerosols from different source regions.

		Aerosol (µg m^−3^)	Na^+^ (µg m^−3^)	NH_4_^+^ (µg m^−3^)	K^+^ (µg m^−3^)	Ca^2+^ (µg m^−3^)	Mg^2+^ (µg m^−3^)	Cl^−^ (µg m^−3^)	NO_3_^−^ (µg m^−3^)	SO_4_^2−^ (µg m^−3^)	[H^+^]_total_ (nmol m^−3^)	∑^+^/∑^−^
**Local**	Fine	34.1	0.4	1.3	1.7	0.10	0.13	2.4	5.1	5.8	104.8	0.59
	Coarse	18.2	1.9	0.08	0.6	0.61	0.45	1.6	1.6	2.1	−57.5	1.51
**Continental**	Fine	44.2	0.9	1.1	1.1	0.26	0.16	1.2	4.1	4.5	39.2	0.79
	Coarse	12.2	0.7	0.05	0.22	0.40	0.35	1.3	1.1	1.8	−2.6	1.03
**Marine**	Fine	9.2	0.7	0.2	0.7	0.03	0.07	0.7	0.8	2.1	−11.3	1.20
	Coarse	26.4	3.9	0.03	0.1	0.2	0.12	3.3	2.1	1.6	8.03	0.96

The higher contribution of local and continental sources to the fine mode aerosol was observed with a little influence of marine sources while the concentration of coarse mode aerosol was found to be higher from the marine source regions than the local and other continental sources. The concentrations of Na^+^ and Cl^−^ follow the same sequence and their coarse mode concentrations were found to be the highest for marine air parcel, indicating the strong influence from the sea. The higher concentrations of fine mode Cl^−^ for continental and local sources indicate the influence of biomass and coal burning. The higher concentrations of coarse mode Ca^2+^ and Mg^2+^ from local and continental sectors indicate the resuspension of local and wind blown soil dust particles. The concentration of coarse mode Mg^2+^ from marine sectors shows the influence of sea salt Mg^2+^. Local and continental sectors show the higher concentrations of fine and coarse mode K^+^ indicating the strong influence of biomass burning and soil dust aerosol respectively. The higher concentrations of fine mode NH_4_^+^, NO_3_^−^ and SO_4_^2−^ from local and continental sectors show a very strong influence of anthropogenic activities whereas coarse mode NO_3_^−^ shows higher concentration from marine sectors. This coarse mode nitrate from marine sectors is not sea salt nitrate rather it indicates the interaction between sea salt aerosol and gas phase HNO_3_ and its association with sodium through chloride displacement reaction discussed earlier. Fine mode aerosol shows higher [H^+^]_total_ and lower Σ^+^/Σ^−^ (104.8 nmol m^−3^ and 0.59, respectively) from the local compared to continental (39.2 nmol m^−3^ and 0.79, respectively) and marine (−11.3 nmol m^−3^ and 1.2, respectively) source regions whereas coarse mode aerosol shows maximum [H^+^]_total_ and minimum Σ^+^/Σ^−^ ratio (8.03 nmol m^−3^ and 0.96, respectively) from marine source compared to local (−57.5 nmol m^−3^ and 1.51, respectively) and continental sources (−2.6 nmol m^−3^ and 1.03, respectively). Thus, local and continental fine mode aerosols were more acidic than coarse mode aerosol whereas marine fine mode aerosols were less acidic compared to coarse mode aerosol.

**Figure 15:**
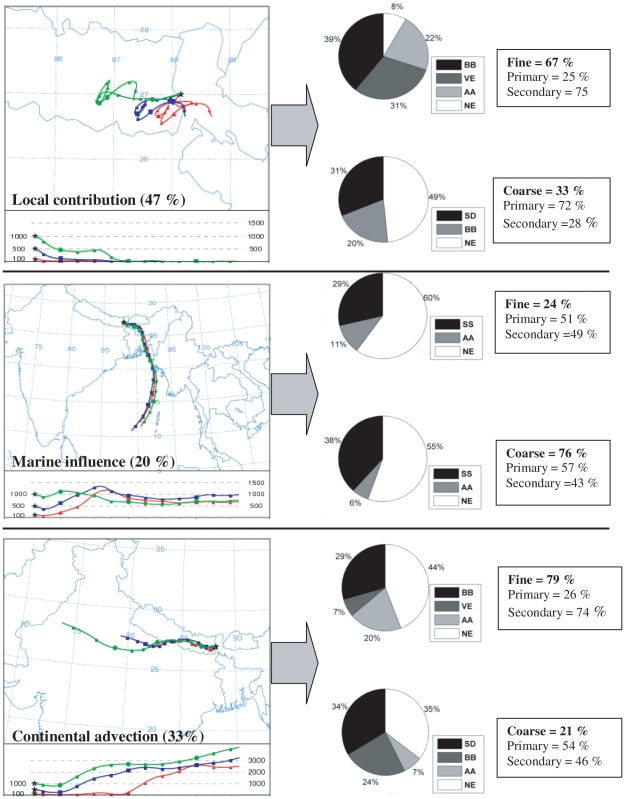
Several source types of aerosol from different source regions. Contributions from local and continental sources (with higher enrichment of fine aerosols) were found to be higher comparative to marine sources (with higher enrichment of coarse aerosols).

Based on the Principal Component Analysis (for source types) and HYSPLIT trajectory model (for source regions) the percentage distribution of several source types of water soluble ions from different source regions (local, continental and marine) in fine and coarse mode aerosol were determined and depicted in Figure 15. In this figure, the percentage distribution of source regions was determined based on the ratio of the number of events of the respective regions (using HYSPLIT) to the total number of events. The figure also describes the percentage distribution of ionic species between primary and secondary species. The percentage of the primary species was determined based on the ratio of sum of the concentrations of chloride, sodium, potassium, calcium and magnesium to the total ionic concentration of aerosol whereas ammonium, nitrate and sulphate were used for the determination of percentage of secondary species in a similar way. Principal component analysis was done using data set from each sector (local, continental and marine) separately for fine and coarse mode aerosol. That means the percentage distribution of various source types (shown in Figure 15) is actually the percentage of variation obtained from factor analysis by PCA.

Figure 15 shows that, 80% of the ionic species originated from the local and other continental sources and 20% was from the marine source. Biomass burning and vehicular emissions (non-sea-potassium, non-sea-sulphate, non-sea-chloride, nitrate) were the major sources for fine mode local and continental aerosols whereas dust particles (non-sea-calcium and magnesium) were the major source for coarse mode local and continental aerosols. On the other hand, the major source for fine and coarse mode marine aerosol was sea salt aerosols enriched mainly with sodium and chloride.

### Conclusions

The major findings of this study can be summarized as:

1. The average concentration of fine mode aerosol is found to be higher than coarse mode aerosol during dry seasons whereas monsoon shows higher loading of coarse mode aerosol compared to fine mode.
2. This study discusses the formation pathways of major secondary aerosol components. Nitrate is found to exist as ammonium nitrate in fine mode aerosol during winter whereas during monsoon nitrate exists as sodium nitrate in coarse mode aerosol. Photochemical oxidation of SO_2_ is the major pathway for the formation of sulphate during premonsoon whereas some other aqueous phase transformation processes are important pathways during winter in sulphate formation.
3. There is a major contribution of non-sea-sulphate and nitrate in the chloride depletion from fine and coarse mode sea salt aerosols, respectively. The chloride depletion, *i.e.* the interaction between marine and urban aerosol, is found to govern the dry removal of nitrate from the atmosphere. The nitrate which is found to be enriched mostly in fine mode during dry seasons gets enriched in coarse mode during monsoon because of the chloride displacement from coarse mode sea salt aerosol. Thus nitrate is found to be deposited (dry or free fall deposition) at a faster rate during monsoon compared to dry seasons.
4. The acidity of fine mode aerosol is found to be higher in dry seasons compared to monsoon whereas the acidity of coarse mode acidity is found to be higher in monsoon compared to dry seasons. Non-sea-sulphate and nitrate are found to govern the acidity of fine mode aerosol whereas non-sea-calcium and non-sea-magnesium govern the coarse mode acidity.
5. Three major source regions like local, continental (mainly from north-western part of India) and marine (Bay of Bengal and Arabian Sea) sources are identified based on HYSPLIT backward trajectory model. Local and other continental source regions contribute 80% with high loading of secondary anthropogenic species whereas the contribution from marine source regions is 20% enriched with primary sea salt particles.
6. Biomass burning and vehicular emissions are the major sources for fine mode local and continental aerosols with higher acidity whereas soil dust particles are the major source for coarse mode local and continental aerosols with lower acidity. On the other hand, the major source for fine and coarse mode marine aerosol is sea salt aerosols with higher acidity in coarse mode.
